# Supplementary material for: A high-resolution mRNA expression time course of embryonic development in zebrafish
Source: eLife. 2017 Nov 16;6:e30860. doi: 10.7554/eLife.30860 (PMC5690287; doi:10.7554/eLife.30860)
Supplement: Supplementary file 6. [file elife-30860-supp6.zip › biolayout-clusters-files/Cluster123-genes.html]

Cluster123


# Cluster123: Genes

| Ensembl ID | Gene Name | Chr | Start | End | Biotype |
| --- | --- | --- | --- | --- | --- |
| ENSDARG00000029848 | FBXO15 | 2 | 23004136 | 23021368 | protein\_coding |
| ENSDARG00000078055 | acd | 7 | 28809534 | 28822820 | protein\_coding |
| ENSDARG00000078825 | bub1ba | 20 | 53669546 | 53704579 | protein\_coding |
| ENSDARG00000023814 | dyrk1ab | 15 | 19948514 | 19961084 | protein\_coding |
| ENSDARG00000013528 | mcm9 | 20 | 41904803 | 41942634 | protein\_coding |
| ENSDARG00000034956 | myclb | 19 | 31218200 | 31223848 | protein\_coding |
| ENSDARG00000076176 | ptcd1 | 1 | 7871212 | 7882456 | protein\_coding |
| ENSDARG00000075834 | si:dkey-182i3.8 | 15 | 23592745 | 23597836 | protein\_coding |
| ENSDARG00000043566 | si:dkey-22a1.3 | 9 | 27570572 | 27581162 | protein\_coding |
| ENSDARG00000079716 | sin3aa | 25 | 6222476 | 6263869 | protein\_coding |
